# Parenting stress and young children’s problematic media use: the serial mediating roles of parental phubbing and parent–child conflict

**DOI:** 10.3389/fpsyg.2026.1806359

**Published:** 2026-04-02

**Authors:** Maoyong Huang, Wen Guo, Ting He, Tengfei Guo, Yakun Ni, Jialing Zhang, Shaoyan Wei, Yanzhen Xu

**Affiliations:** 1 Guangdong Polytechnic Normal University, Guangzhou, China; 2 Guangdong University of Finance, Guangzhou, China

**Keywords:** parental phubbing, parent–child conflict, parenting stress, preschool children, problematic media use

## Abstract

**Background:**

Excessive screen media use among young children has become a growing public health concern. This study aimed to examine a sequential pathway in which parenting stress influences young children’s problematic media use (PMU) by first increasing parental phubbing, which then exacerbates parent–child conflict.

**Methods:**

This study employed a multi-wave design. Data were collected via an online questionnaire survey from 925 parents of children aged 3–6 years in Guangdong Province, China. Participants completed the Parenting Stress Index-Short Form (PSI-SF), the Generic Scale of Phubbing (GSP), the Parent–Child Conflict Scale (PCC), and the Children’s Problematic Media Use Questionnaire (CEMUQ). The mediation analysis was conducted using Hayes’s PROCESS macro (Model 6) with 5,000 bootstrap samples.

**Results:**

Parenting stress was significantly positively associated with children’s PMU. This relationship was independently mediated by both parental phubbing and parent–child conflict. More importantly, a significant serial mediation path was confirmed: parenting stress increased parental phubbing, which in turn heightened parent–child conflict, ultimately leading to higher levels of PMU.

**Conclusion:**

This study confirms that parenting stress affects young children’s PMU by increasing parental phubbing, which subsequently aggravates parent–child conflict. These findings suggest that effective intervention requires a dual focus on alleviating parenting stress and improving parent–child interaction quality.

## 1 Introduction

With the rapid advancement of digital technology and the ubiquity of smart devices, problematic media use (PMU) among young children has emerged as a growing public health concern. PMU refers to the uncontrollable overuse of screen-based devices, accompanied by impairments in social, behavioral, and cognitive functioning—such as persistent device-seeking behaviors, emotional dysregulation when screen time is restricted, or reduced engagement in developmental tasks (Domoff et al., 2020; Coyne et al., 2021). In recent years, the prevalence of early childhood screen exposure has increased substantially. For instance, in both China and the United States, preschoolers’ daily screen time frequently exceeds the recommended two-hour limit (Hua et al., 2022; Rideout and Robb, 2017; Domoff et al., 2019), a trend that has become even more pronounced in the post-pandemic era (Gueron-Sela et al., 2023). Similarly, in countries like South Korea, children aged three to five spend an average of up to 4 h per day on digital devices (Kim et al., 2020). Although not all forms of screen exposure are inherently harmful, such pervasive and early media engagement markedly increases the risk of young children transitioning from normative to problematic users (Fitzpatrick et al., 2025). Given the significance of this phenomenon, it is important to identify factors within the family environment that drive the progression from high-frequency media exposure to PMU.

Parenting stress, defined as the negative emotional experience parents face when their resources are insufficient to meet parenting demands, has garnered increasing attention (Brauchli et al., 2024; Abidin and Brunner, 1995). High levels of parenting stress can significantly deplete parents’ patience and self-regulation, leading to emotional exhaustion. To cope with this internal depletion, parents are more likely to resort to less effortful and passive parenting strategies. Specifically, when a child cries or seeks attention, the use of smartphones or tablets as “electronic babysitters” to soothe the child becomes a common coping mechanism, providing a brief respite (Radesky et al., 2016; McDaniel and Radesky, 2020). When such behavioral patterns recur, they may deprive young children of crucial opportunities to learn co-regulation of emotions with their parents. More critically, such behavioral patterns may reinforce young children’s maladaptive perception of electronic devices as effective tools for soothing negative emotions and alleviating boredom. This process establishes the foundation for media use to be internalized as a primary emotion regulation strategy, thereby directly increasing the risk of PMU.

According to family systems theory, parental behavior plays a pivotal role in shaping parent–child interaction patterns (Cox and Paley, 2003). Within this framework, the spillover effect posits that an individual’s emotional and stress states can permeate dyadic interactions and subsequently compromise relationship quality (Sears et al., 2015). Parenting stress may not only drive parents to utilize media as “electronic babysitters,” but also indirectly influence young children’s behaviors through the more concealed mechanism of altering parents’ own media usage habits. In high-stress situations, many parents themselves turn to smartphones for relaxation or escapism, a behavior that manifests in parenting interactions as “parental phubbing” (Griffith, 2023). Phone neglect is not merely a distraction, but a profound emotional withdrawal. For instance, when a child excitedly presents a block structure, a parent absorbed in their phone may offer delayed or dismissive responses, or even no response at all. This lack of immediate feedback and emotional resonance communicates signals of neglect and unimportance to the child (Kildare and Middlemiss, 2017).

Viewed through the lens of the spillover effect, stress-induced avoidant media use fundamentally diminishes parental emotional responsiveness and sensitivity, which in turn alters interaction patterns. Persistent emotional withdrawal can be interpreted as a mild yet repetitive form of emotional neglect, a core subtype of emotional child maltreatment (Lyons-Ruth and Yarger, 2022; Shaffer et al., 2009). Although rarely driven by malice, the repetitive nature of parental phubbing inadvertently heightens the risk of young children experiencing emotional neglect (Glaser, 2002). Such emotional detachment and neglect may lead the child to seek comfort in electronic media, gradually developing a reliance on it (Chotpitayasunondh and Douglas, 2016). Research has shown that when parents are engrossed in their phones during meals, play, or outings, children often exhibit more negative emotions, such as anger, anxiety, or aggression, as they perceive an emotional deficit in the parent–child relationship (Kildare and Middlemiss, 2017). Furthermore, parental phone neglect not only reduces the frequency of responding to children’s needs but also weakens the emotional connection between parent and child, subsequently affecting the child’s emotional attachment (Domoff et al., 2018). In such contexts, young children may turn to electronic media as a substitute source of emotional comfort, thereby exacerbating their dependency on media use.

Furthermore, parenting stress may also influence young children’s media usage by increasing the frequency of parent–child conflicts, representing a sequential extension of the aforementioned spillover process. Parent–child conflict typically manifests as poor communication, emotional confrontation, or behavioral opposition. Parents experiencing emotional depletion have significantly reduced patience and empathy, making them more likely to misinterpret a child’s normal behavior of seeking attention as unreasonable demands. For example, a simple request such as “play with me” or “read with me” from a young child may be perceived by an exhausted parent as an unwarranted nuisance, rather than a desire for emotional connection. The impatient rejection by the parent is likely to trigger the child’s crying or defiance, which, in turn, reinforces the parent’s view that the child is difficult to manage, thereby drawing both parties into a negative interaction cycle (Pianta, 1997). In accordance with the Family Stress Model, emotional dysregulation under high stress not only affects individual behavior but also undermines relationship quality and generates these negative interaction cycles. In this persistently tense family environment, the parent–child relationship, which should provide emotional support for the child, instead becomes a source of stress for the child. As a result, young children are more inclined to seek out media (e.g., watching cartoons or playing mobile games) as a cognitive and emotional avoidance strategy, using it to “escape” from the conflict-ridden reality and gain immediate emotional relief (Hale and Guan, 2015). Recent empirical evidence has confirmed that significantly increased media usage duration and frequency are observed among young children exposed to chronic, high-intensity parent–child conflicts, indicating media internalization as a primary alternative emotion regulation strategy (Al-Shoaibi et al., 2024; Gu and Zhao, 2025).

**Figure 1:**
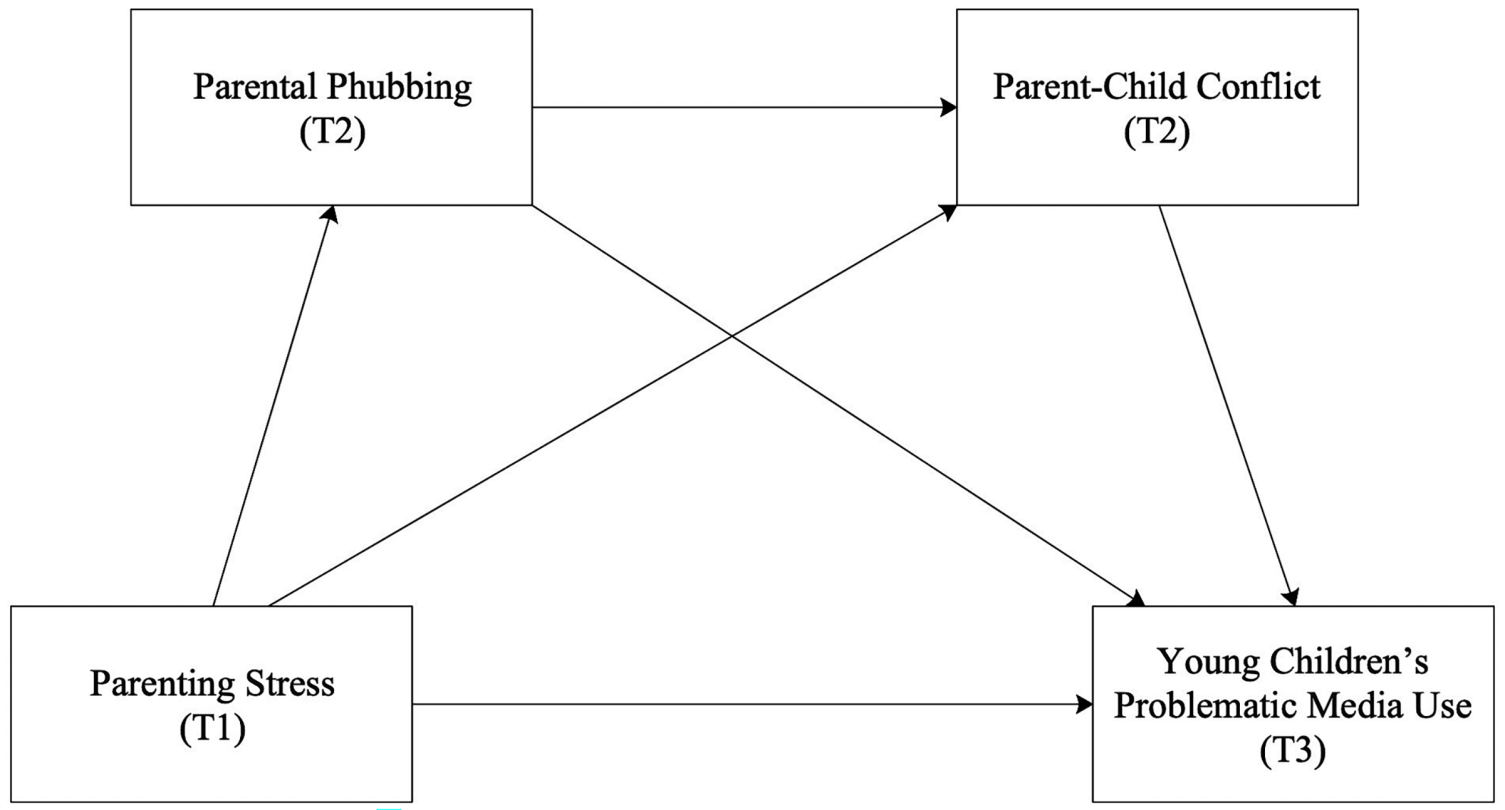
Theoretical model.

The present study aims to investigate the specific mechanisms linking parenting stress to children’s PMU. Guided by family systems theory and the family stress model, this research proposes that parenting stress influences young children by increasing parental phubbing and exacerbating parent–child conflict. This influence operates through a serial chain reaction: parents under stress are more inclined to seek escapism through smartphone use, leading children to perceive a form of invisible emotional neglect. This neglect weakens emotional attunement and intensifies parent–child conflict, ultimately prompting children to rely on media for emotion regulation (Cox and Paley, 1997; Hale and Guan, 2015). Given that previous studies have predominantly relied on cross-sectional designs, they struggle to establish temporal precedence or exclude reciprocal effects, such as children’s PMU reinforcing parenting stress. To address these limitations, this study employs a multi-wave data collection design, measuring the predictor, mediators, and outcome variables at distinct time points. This separated measurement approach helps mitigate common method bias and provides a more rigorous verification of the developmental sequence from stress spillover to behavioral outcomes. This study contributes to the extant literature in three ways: first, it illuminates how parental behaviors under stress impact children’s media dependency through the quality of family interactions; second, it identifies the critical role of parental phubbing in disrupting interactive attunement; and finally, it elucidates parent–child conflict as a mediating mechanism within the stress spillover process. These perspectives offer novel theoretical insights into the impact of family stress in the digital era (Figure 1).

## 2 Literature review and hypothesis development

### 2.1 Parenting stress and young children’s problematic media use

Parenting stress refers to the aversive psychological reaction to the demands of being a parent, often triggered by a mismatch between parenting resources and demands (Abidin and Brunner, 1995). According to the family stress model, external stressors such as economic hardship are first translated into parents’ internal psychological distress, of which parenting stress is a primary component (Abidin and Brunner, 1995; Crnic et al., 2005). This negative experience may alter parental practices, leading to less responsive or maladaptive parenting (Scrimin et al., 2022). Specifically, negative parenting behaviors manifest in two distinct pathways. First, parents may rely on electronic devices as low-effort tools to manage children’s behavior (Scrimin et al., 2022). Second, parents’ own media use may serve as a strategy to escape stress, which inadvertently displaces parent–child interactions and models problematic media habits (McDaniel and Radesky, 2020). Collectively, these behaviors increase children’s media exposure and the risk of media dependency (Putnick et al., 2023). Recent empirical evidence supports this link, indicating a significant association between parenting stress and PMU among children aged 3–6 years (Kattein et al., 2023). Higher levels of parental distress and general stress intensify family tension, prompting children to seek media as an emotional escape. This process, in turn, contributes to excessive media consumption (Duch et al., 2013; McDaniel and Radesky, 2020; Nathanson and Manohar, 2012; Pempek and McDaniel, 2016). In line with the principle of falsification, the following alternative hypothesis is formulated to be tested against the null hypothesis:

*H1*: Parenting stress is positively related to young children’s PMU.

### 2.2 The mediating role of parental phubbing

In the digital era, the widespread use of smartphones has given rise to a new social phenomenon known as “phubbing.” The term, a portmanteau of “phone” and “snubbing,” refers to the act of ignoring others in social interactions due to one’s preoccupation with mobile devices (Chotpitayasunondh and Douglas, 2016; Karadağ et al., 2016). When such behavior occurs in parent–child contexts, it constitutes parental phubbing, characterized by parents’ neglect of children’s emotional needs and interactional cues due to their immersion in mobile phone use (Ding et al., 2018; Jiang et al., 2021).

Within the framework of family systems theory, parenting stress is regarded as an external factor that profoundly shapes family dynamics. Parents experiencing high levels of stress often display more negative behavioral patterns during parent–child interactions. Parental phubbing, as a typical manifestation of negative interaction in the family environment, is defined by the prioritization of mobile media over the child, leading to reduced nonverbal engagement and disrupted emotional communication (Radesky et al., 2015). Elevated parenting stress may weaken parents’ emotion regulation, resulting in frustration and helplessness during childcare. These negative emotions undermine nonverbal communication, as parents become less responsive to children’s eye contact, touch, and facial expressions (Karadağ et al., 2016). Concurrently, heightened stress may drive parents to engage with digital media as a means of temporary emotional relief. Immersion in digital spaces allows parents to momentarily escape parenting burdens, yet such behavior diminishes parental attention and deepens emotional disconnection. When parental focus is absorbed by mobile devices, children’s emotional needs often go unnoticed, resulting in reduced emotional responsiveness and increased relational distance (Kim et al., 2015).

Essentially, this stress-induced phubbing reflects emotional withdrawal by the parent. In developmental psychology, such persistent behavior is regarded as a mild but repetitive form of emotional neglect. As an implicit subtype of child maltreatment, emotional neglect occurs when parents fail to respond timely and appropriately to a child’s needs. This failure weakens secure attachment and hinders the development of emotion regulation in young children (Glaser, 2002; Kim and Cicchetti, 2010; Norman et al., 2012). When parents focus on screens rather than their children, the child frequently experiences feelings of exclusion. Although this subtle harm is often overlooked, it has long-term negative effects on social–emotional development (Zhang et al., 2023). Research indicates that stressed parents are more likely to use digital media to regulate their own emotions (Wadley et al., 2020). While this provides short-term relief, habitual phubbing significantly reduces parental sensitivity to the child’s needs (McDaniel and Coyne, 2016). Since emotional neglect is closely linked to various behavioral problems, children lacking stable emotional support often turn to digital media for alternative companionship and comfort (Ogundele, 2018). When real-life interactions fail to provide sufficient emotional feedback, children develop an emotional reliance on media, which eventually evolves into problematic media use (Domoff et al., 2019; Jusienė et al., 2025).

Furthermore, parental phubbing may activate children’s social learning mechanisms. According to social learning theory, parents serve as primary behavioral models for young children (Niu et al., 2020). Frequent parental engagement with digital devices implicitly conveys the value that media interactions are more rewarding than interpersonal communication and models media use as the preferred leisure activity (Poulain et al., 2019). Through observation and imitation, children are likely to internalize these behavioral tendencies, which, in turn, escalate their own media use (Schwarzer et al., 2022). Previous empirical studies have demonstrated that higher parenting stress is associated with increased parental phubbing, which subsequently contributes to children’s PMU (Radesky et al., 2016). Therefore, the following alternative hypothesis is proposed:

*H2:* Parental phubbing mediates the relationship between parenting stress and young children’s PMU.

### 2.3 The mediating role of parent–child conflict

Parent–child conflict refers to a negative dimension of the parent–child relationship, characterized by adverse interactions, frequent arguments, emotional confrontations, and communication barriers (Pianta, 1997). These conflicts contribute to substantial tension and emotional distance within the family system. According to the Family Stress Model (Conger et al., 2002), parenting stress acts as a catalyst for such conflict by eroding parental patience and emotional availability. Specifically, parents experiencing high stress are more likely to adopt maladaptive coping strategies such as avoidance or excessive control. Instead of supportive communication, they may respond to children’s behaviors with criticism or punishment (Deater-Deckard and Petrill, 2004). Consequently, parenting stress intensifies intrafamilial conflict, transforming the family microsystem—which typically provides security—into an unpredictable and threatening environment (Bronfenbrenner, 1979).

According to family systems theory, such conflictual environments significantly hinder children’s socio-emotional development and induce anxiety (Morelli et al., 2022; Yang et al., 2022). To escape the distress associated with family tension and compensate for the lack of emotional support, children may seek alternative means of emotion regulation (Hale and Guan, 2015). Digital media, with its immersive and responsive features, provides a temporary avenue for emotional relief, allowing children to withdraw from stressful real-life interactions (Needlman, 2011). Recent empirical studies have confirmed that poor parent–child relationships contribute to maladaptive media use (Wong et al., 2020), and high-conflict family environments increase children’s reliance on electronic devices to manage emotional distress (Hale and Guan, 2015). Based on the above analysis, the following alternative hypothesis is proposed:

*H3*: Parent–child conflict mediates the relationship between parenting stress and young children’s PMU.

### 2.4 The sequential mediating effects of parental phubbing and parent–child conflict

Family systems theory emphasizes the interdependence of family members, positing that changes in one member’s behavior reverberate through the entire family system (Cox and Paley, 1997). Building upon the spillover effect within this framework, we propose a serial mediation pathway where individual psychological distress first manifests as behavioral withdrawal and subsequently impairs the dyadic relational climate. First, chronic parenting stress may drive parents to use mobile devices as a coping mechanism to escape emotional distress, thereby fostering habitual dependency (Coyne et al., 2014). This behavioral shift fundamentally disrupts family dynamics. Specifically, as parents direct more attention toward their phones, the quality of parent–child interactions deteriorates, exacerbating emotional detachment. When children’s bids for attention are repeatedly thwarted by parental phubbing, children may escalate their behaviors (e.g., crying or disruptive actions) to elicit attention. Distracted parents often misinterpret these escalations as behavioral problems, responding with impatience or reprimands. Such negative cycles accumulate into parent–child conflict (Kildare and Middlemiss, 2017). Empirical evidence supports this link, showing that frequent parental phubbing decreases responsiveness, leading to children’s needs being ignored and negatively impacting relationship quality (McDaniel et al., 2018).

In turn, children’s behaviors are deeply shaped by these conflictual interaction patterns. In high-conflict environments, children may cope with emotional deprivation and relational tension by imitating their parents’ behaviors (Cox and Paley, 1997). To compensate for emotional neglect, children may resort to excessive digital media use as a solitary refuge. Based on this analysis, we infer that parenting stress not only contributes to parental phubbing but may also, through the intensification of parent–child conflict, jointly influence children’s PMU. Therefore, the following alternative hypothesis is proposed:

*H4*: Parental phubbing and parent-child conflict sequentially mediate the relationship between parenting stress and young children’s PMU.

## 3 Materials and methods

### 3.1 Participants and procedures

Data collection was conducted via an online questionnaire platform across eight prefecture-level cities in Guangdong Province, including Guangzhou, Foshan, Zhuhai, Jiangmen, Qingyuan, Heyuan, Shanwei, and Shantou. To ensure representativeness, a stratified random sampling strategy was adopted. First, regional stratification was conducted based on economic development disparities between the Pearl River Delta and the northern and eastern Guangdong regions. Second, within each city, a secondary stratification was performed based on kindergarten type (public vs. private). Ultimately, 43 kindergartens were selected as collaborating institutions. Eligibility criteria were strictly established to align with the research variables: (a) children aged 3 to 6 years; (b) families owning at least two smart electronic devices (e.g., smartphones or tablets); and (c) parents identifying as the primary caregiver. Single-parent households and those with grandparents as primary caregivers were excluded to control for family structure and caregiving consistency.

In terms of procedure, the research team distributed survey links via parent contact groups established by each kindergarten. The landing page detailed the study’s purpose, instructions, and privacy statement. To enhance construct validity, the questionnaire provided specific contextual examples for items like “parenting stress” and “phubbing behavior” (e.g., “When your child is noisy, have you ever given them a phone to gain peace?”). Furthermore, a built-in logic check system was employed to prompt a review if contradictory responses were detected (e.g., choosing both “never use a phone” and “frequently have conflicts with children over phone use”).

To mitigate potential common method bias (CMV), data collection involved three waves of surveys administered at four-week intervals. At the initial time-point (T1), a web link was distributed to 1,150 parents. The survey assessed parenting stress and demographic variables. After excluding incomplete responses and outliers, 1,062 valid questionnaires were retained, yielding a response rate of 92.34%. Four weeks later, at the second time-point (T2), the same 1,062 participants were invited to assess parental phubbing and parent–child conflict, resulting in 971 valid responses and a response rate of 91.43%. At the third time-point (T3), participants were asked to evaluate their children’s problematic media use (PMU). Ultimately, 925 complete and matched responses were collected, achieving a final response rate of 95.26%.

The final sample comprised 925 parents (42.5% fathers, 57.5% mothers). Regarding parental age, 44.5% were 30 or younger, 47.2% were 31–40, and 8.2% were 41 or older. In terms of educational attainment, 46.7% had a junior college degree or below, 39.1% held a bachelor’s degree, and 14.2% held a master’s degree or higher. The distribution of monthly family income was as follows: 17.5% earned 5,000 RMB or less, 53.8% earned 5,001–9,999 RMB, and 28.6% earned 10,000 RMB or more. Children’s grade levels were distributed as follows: 31.8% in Junior Class (aged 3–4 years), 35.9% in Middle Class (aged 4–5 years), and 32.3% in Senior Class (aged 5–6 years). In terms of gender, 56.0% were boys and 44.0% were girls. These data confirm the sample’s diversity and representativeness, effectively capturing parenting and media use patterns across varying socioeconomic contexts in Guangdong Province.

### 3.2 Measures

#### 3.2.1 Young children’s problematic media use

Young children’s PMU was assessed using the Children’s Electronic Media Use Questionnaire adapted by Li (2018). The questionnaire consists of 20 items across four dimensions: Time Management (4 items; e.g., “Does your child use electronic media for longer than originally planned?”), Interpersonal and Health (4 items; e.g., “Does your child appear more fatigued or lethargic after using electronic media?”), Life Conflict (5 items; e.g., “Has your child reduced outdoor play due to electronic media use?”), and Emotional Experience (7 items; e.g., “Does your child use electronic media when feeling bad or upset?”). Items were rated on a five-point Likert scale (1 = never, 5 = always), with higher scores indicating greater dependence on media. In the present study, the Cronbach’s *α* coefficient for the total scale was 0.921. For the subscales of Time Management, Interpersonal and Health, Life Conflict, and Emotional Experience, the Cronbach’s *α* coefficients were 0.739, 0.761, 0.759, and 0.836, respectively. Confirmatory factor analysis (CFA) indicated a good model fit: *χ^2^* = 171.598, *df* = 146, *p* = 0.073, CFI = 0.996, TLI = 0.995, RMSEA = 0.014, and SRMR = 0.019.

#### 3.2.2 Parenting stress

Parenting stress was measured using the Parenting Stress Index-Short Form (PSI-SF) developed by Luo et al. (2021). The scale consists of 36 items distributed across three dimensions: Parenting Distress, Parent–Child Relationship Dysfunction, and Difficult Child (12 items per dimension). Parenting Distress refers to parents’ personal issues, such as depression or lifestyle limitations due to parenting demands; Parent–Child Relationship Dysfunction reflects parents’ satisfaction with their interactions and their level of acceptance of the child; Difficult Child pertains to parents’ views on their child’s self-regulation. Responses were recorded on a five-point Likert scale (1 = strongly disagree, 5 = strongly agree), with higher scores indicating higher levels of parenting stress. The Cronbach’s *α* coefficient for the total scale was 0.955, and for the subscales, coefficients were 0.904, 0.906, and 0.895, respectively. The CFA results indicated an acceptable model fit: *χ ^2^* = 832.976, *df* = 591, *p* < 0.001, CFI = 0.983, TLI = 0.982, RMSEA = 0.021, and SRMR = 0.022.

#### 3.2.3 Parental phubbing

Parental phubbing was measured using the Generic Scale of Phubbing developed by Chotpitayasunondh and Douglas (2018). This scale comprises 15 items across four dimensions: Mobile Phone Anxiety (4 items; e.g., “Do you feel anxious when your phone is not with you?”), Interpersonal Conflict (4 items; e.g., “Do you have conflicts with others due to phone use?”), Self-Isolation (4 items; e.g., “Would you prefer focusing on your phone rather than talking to others?”), and Problematic Awareness (3 items; e.g., “Do you spend more time on your phone than you intended?”). Items were rated on a five-point Likert scale (1 = strongly disagree, 5 = strongly agree), with higher scores indicating more severe phubbing behavior. The Cronbach’s α coefficient for the total scale was 0.889. For the subscales of Mobile Phone Anxiety, Interpersonal Conflict, Self-Isolation, and Problematic Awareness, the Cronbach’s α coefficients were 0.761, 0.736, 0.708, and 0.658, respectively. The CFA results demonstrated an acceptable model fit: *χ*^2^ = 119.819, *df* = 84, *p* < 0.01, CFI = 0.991, TLI = 0.989, RMSEA = 0.021, and SRMR = 0.021.

#### 3.2.4 Parent–child conflict

The Parent–Child Conflict Scale, derived from Pianta (1992), was used to assess conflict within the parent–child relationship. This unidimensional scale consists of 12 items evaluating emotional connection, communication, and conflict levels (e.g., “My child and I seem to be constantly competing with each other”). Responses were rated on a five-point Likert scale (1 = strongly disagree, 5 = strongly agree), where higher scores indicate greater conflict. In this study, the Cronbach’s α coefficient for the scale was 0.885. The CFA results showed a good model fit: *χ*^2^ = 78.907, *df* = 54, *p* < 0.05, CFI = 0.993, TLI = 0.991, RMSEA = 0.022, and SRMR = 0.020.

#### 3.2.5 Control variables

Demographic variables were included as covariates, consisting of parents’ gender, age, education level, and monthly household income, as well as child gender and grade. Parental and family characteristics were controlled based on prior evidence linking them to parenting styles and media resource availability (Du et al., 2022; Gebremariam et al., 2020; Negi and Sattler, 2026; Tang et al., 2018). Additionally, child gender and grade (serving as a proxy for age) were included to account for established developmental and gender differences in children’s media usage patterns (Domoff et al., 2019; Lauricella et al., 2015).

### 3.3 Data analysis

The statistical analyses were conducted using IBM SPSS Statistics 29.0. Prior to hypothesis testing, preliminary analyses were performed, including tests for common method bias and reliability analysis. Descriptive statistics and Pearson correlation analyses were then conducted to examine the basic relationships among the study variables. Parent gender, parent age, education level, family monthly income, child gender, and child grade were included as control variables in subsequent analyses. All statistical tests were two-tailed, and the significance level was set at *p* < 0.05. To test the hypotheses, hierarchical regression analysis was first conducted to examine the direct effect of parenting stress on young children’s PMU. Building on this analysis, a serial mediation model was estimated through the utilization of Model 6 within the PROCESS macro (Hayes, 2017). Model 6 was selected because the study hypothesized that parental phubbing and parent–child conflict operate as sequential mediators linking parenting stress to children’s PMU. The mediation effects were tested using a bias-corrected bootstrap procedure with 5,000 resamples to generate 95% confidence intervals (CI). An indirect effect was considered statistically significant when the confidence interval did not include zero. To provide standardized interpretations of the results, standardized regression coefficients (*β*) and model explanatory power (*R^2^*) were reported as indicators of effect size. Cohen’s guidelines were adhered to in the investigation of effect size magnitudes (Cohen, 1988), ranging between small (0.10), medium (0.30), and large effects (0.50).

## 4 Results

### 4.1 Common method bias test

Since the data in this study were all collected through self-report methods, in order to control for potential common method bias, the study implemented several procedural measures, including anonymous completion, adjusting the order of the scales, and setting reverse-coded items. On this basis, Harman’s single-factor method (Podsakoff et al., 2003) was used for post-hoc statistical testing. The results of the test indicated that nine factors with eigenvalues greater than 1 were extracted from the data, and the maximum variance explained by the first common factor was 21.58%, which did not exceed the 40% threshold. Based on this, it can be concluded that there is no significant common method bias in this study.

### 4.2 Descriptive statistics and correlation analysis

Demographic variables, including parents’ gender, age, education level, and family monthly income, as well as child gender and child grade, were set as control variables. Table 1 presents the correlations among the variables. Parenting stress, parental phubbing, parent–child conflict, and children’s PMU were all found to be significantly correlated. Specifically, children’s PMU was significantly and positively correlated with parenting stress (*r* = 0.315, *p* < 0.001), parental phubbing (*r* = 0.236, *p* < 0.001), and parent–child conflict (*r* = 0.279, *p* < 0.001), providing a preliminary basis for the subsequent mediation analysis.

**Table 1:** Means, standard deviations, and correlations.

Variable	*M*	SD	1	2	3	4	5	6	7	8	9	10
1. Parent gender	1.58	0.50	–									
2. Parent age	1.64	0.63	0.011	–								
3. Education	1.67	0.71	−0.009	0.045	–							
4. Income	2.11	0.67	0.032	0.029	0.085^**^	–						
5. Child gender	1.44	0.50	0.035	0.062	0.001	−0.027	–					
6. Child grade	2.01	0.80	−0.06	0.008	0.037	0.051	0.032	–				
7. Parenting stress	2.98	0.77	−0.033	0.019	0.01	0	0.117^**^	0.001	–			
8. Parental phubbing	3.03	0.81	−0.001	−0.015	0.070^*^	−0.006	0.106^**^	−0.048	0.318^***^	–		
9. Parent–child conflict	3.04	0.89	0.004	0.005	0.034	−0.012	0.060	−0.018	0.363^***^	0.367^***^	–	
10. Problematic media use	3.12	0.82	−0.023	0.003	−0.176^**^	0.062	0.021	−0.004	0.315^***^	0.236^***^	0.279^***^	–

*N* = 925. *p < 0.05, **p < 0.01, ***p < 0.001.

### 4.3 Testing the hypotheses

To test Alternative Hypothesis 1 (H1), which posited a positive relationship between parenting stress and young children’s PMU, a hierarchical regression analysis was conducted. As shown in Table 2, parenting stress was significantly and positively associated with PMU (*β* = 0.317, *t* = 10.27, *p* < 0.001). According to Cohen’s guidelines, standardized regression coefficients (*β*) of 0.10, 0.30, and 0.50 represent small, moderate, and large effects, respectively (Cohen, 1988). Thus, the observed coefficient indicates a moderate effect, suggesting that parenting stress plays a meaningful role in predicting young children’s PMU. Consequently, the null hypothesis of no association was rejected, providing empirical evidence in favor of H1.

**Table 2:** Regression analysis results (𝑁 = 925).

Predictor variable	Problematic media use		Parental phubbing		Parent–child conflict		Problematic media use	
	*β*	*t*	*β*	*t*	*β*	*t*	*β*	*t*
Parent gender	−0.016	−0.516	0.005	0.164	0.016	0.506	−0.019	−0.636
Parent age	0.005	0.152	−0.027	−0.881	−0.004	−0.132	0.009	0.291
Education	−0.186	−6.044^***^	0.070	2.247	0.033	1.062	−0.200	−6.657^***^
Income	0.078	2.522^*^	−0.006	−0.200	−0.014	−0.455	0.081	2.692^***^
Child gender	−0.014	−0.441	0.073	2.311	0.018	0.567	−0.026	−0.844
Child grade	−0.002	−0.064	−0.052	−1.668	−0.019	−0.604	0.008	0.251
Parenting stress	0.317	10.272^***^	0.310	9.880^***^	0.361	11.665^***^	0.220	6.676^***^
Parental phubbing							0.124	3.746^***^
Parent–child conflict							0.164	4.893^***^
*R*	0.371		0.337		0.366		0.429	
*R* ^2^	0.138		0.114		0.134		0.184	
*F*	20.905^***^		16.837^***^		20.252^***^		22.984^***^	

Standardized coefficients (β) are reported. *p < 0.05, **p < 0.01, ***p < 0.001.

Alternative Hypothesis 2 proposed that parental phubbing mediates the relationship between parenting stress and young children’s PMU. The results in Table 2 indicate that parenting stress was positively related to parental phubbing (*β* = 0.310, *t* = 9.88, *p* < 0.001), representing a moderate effect. When parental phubbing was included in the full model, it was significantly and positively related to PMU (*β* = 0.124, *t* = 3.75, *p* < 0.001), indicating a small effect. After including the mediator, the direct effect of parenting stress on PMU remained significant (*β* = 0.220, *t* = 6.68, *p* < 0.001), showing a small-to-moderate effect. Bootstrap analysis further revealed that the indirect effect via parental phubbing was significant (Effect = 0.036, 95% CI [0.013, 0.061]; see Table 3). As the 95% confidence interval did not encompass zero, the null hypothesis stating the indirect effect equals zero was rejected. This corroborates H2, indicating that parental phubbing partially mediates the relationship. This indirect pathway accounted for 10.75% of the total effect.

**Table 3:** Bias-corrected bootstrap analysis for mediation effects.

	Effect	SE	LLCI	ULCI	Relative effect
Total effect	0.335	0.033	0.269	0.400	–
Direct effect	0.237	0.036	0.167	0.307	–
Total indirect effect	0.098	0.020	0.059	0.138	29.25%
PS → PP → PMU	0.036	0.012	0.013	0.061	10.75%
PS → PCC → PMU	0.046	0.012	0.025	0.073	13.73%
PS → PP → PCC → PMU	0.015	0.005	0.007	0.025	4.48%

Unstandardized coefficients are reported. PS, parenting stress; PP, parental phubbing; PCC, parent–child conflict; PMU, problematic media use. Bootstrapping sample size = 5,000.

To address Alternative Hypothesis 3, the mediating role of parent–child conflict was examined. As shown in Table 2, parenting stress was significantly and positively associated with parent–child conflict (*β* = 0.361, *t* = 11.67, *p* < 0.001), representing a moderate-to-relatively strong effect. Parent–child conflict was also significantly and positively related to PMU (*β* = 0.164, *t* = 4.89, *p* < 0.001), indicating a small-to-moderate effect. Bootstrap results further demonstrated that the indirect effect through parent–child conflict was significant (Effect = 0.046, 95% CI [0.025, 0.073]; see Table 3). The exclusion of zero from the confidence interval allowed for the rejection of the null hypothesis, thereby providing statistical support for H3. This pathway accounted for 13.73% of the total effect. These results suggest that higher parenting stress may increase the likelihood of parent–child conflict, which in turn contributes to children’s PMU. Therefore, Hypothesis 3 was supported.

Finally, Alternative Hypothesis 4 examined the sequential mediation of parental phubbing and parent–child conflict. Bootstrap analysis revealed a significant serial mediation pathway (Effect = 0.015, 95% CI [0.007, 0.025]; see Table 3). Because the confidence interval did not include zero, the null hypothesis that the serial indirect effect equals zero was rejected. Although the magnitude of this effect was relatively small, this serial pathway accounted for 4.48% of the total effect, indicating that parenting stress may influence children’s PMU through a sequential process in which increased parental phubbing triggers parent–child conflict, ultimately contributing to higher levels of PMU. Thus, Hypothesis 4 was supported.

Overall, the total effect of parenting stress on young children’s PMU was found to be accounted for by the three mediating pathways, including parental phubbing, parent–child conflict, and the serial mediation pathway, which together explained 29.25% of the total effect. These results indicate that the association between parenting stress and children’s PMU is influenced by family interaction processes.

## 5 Discussion

### 5.1 Theoretical implications

To address limitations inherent in prior cross-sectional research, the present study made a distinctive contribution by employing a multi-wave longitudinal design, through which the temporal sequencing of mechanisms linking family stress to children’s media dependency was delineated. Specifically, the serial mediating roles of parental phubbing and parent–child conflict were examined in the association between parenting stress and young children’s PMU. The findings indicated that parenting stress was not only directly associated with heightened levels of children’s PMU, but was also indirectly linked to children’s media dependency through systematic disruptions in the quality of family interactions.

First, a direct association was observed between higher levels of parenting stress and elevated children’s PMU. This pattern is consistent with prior evidence suggesting that parental psychological distress constitutes a salient predictor of children’s media-related difficulties (Kattein et al., 2023; McDaniel and Radesky, 2020; Padilla-Walker et al., 2020). In accordance with the Family Stress Model (Conger et al., 1992; Masarik and Conger, 2017), the current results further suggested that heightened parenting stress may be accompanied by systematic depletion of parents’ cognitive and emotional resources. Such resource depletion is likely to undermine parents’ capacity to sustain stable and patient caregiving, thereby weakening their ability to monitor and regulate children’s media use. Under conditions of elevated stress, digital devices may be intentionally deployed as temporary substitutes to reduce parental emotional strain or to manage children’s behavior, which may gradually foster children’s reliance on media (Radesky et al., 2016). Collectively, these findings imply that intrafamilial stress processes may shape children’s media use not only by altering interaction patterns, but also through behavioral transmission; accordingly, interventions solely targeting children’s screen time may be insufficient, whereas reductions in parents’ psychological burden may represent a more central leverage point.

Second, the mediating role of parental phubbing in the association between parenting stress and young children’s PMU was supported. When elevated stress is experienced, parents may be more likely to seek short-term affective relief through immersion in digital media (McDaniel and Radesky, 2018). Such avoidant engagement should not be construed merely as transient attentional diversion; rather, it may function as a subtle form of emotional unavailability that disrupts the synchrony of parent–child interactions (Domoff et al., 2018; Kildare and Middlemiss, 2017). From the child’s perspective, parents’ preoccupation with mobile phones may implicitly communicate the mistaken message that “the device matters more than I do,” which may prompt children to turn to the virtual world to obtain a sense of belonging and predictability that is perceived as lacking in offline interactions (Kim et al., 2015). In addition, parents’ stress-related, avoidant media use may not only destabilize relational equilibrium, but may also exert a robust modeling effect, through which digital media are implicitly framed as tools for emotion management and for compensating emotional voids (Suh et al., 2024).

Third, the mediating effect of parent–child conflict provided additional evidence that deterioration in the family emotional climate constitutes a critical pathway through which children’s media dependency may be intensified. Consistent with the Family Stress Model (Shao et al., 2024), increased emotional tension and interpersonal friction within the family are theorized to erode children’s sense of emotional security, thereby motivating compensatory efforts to seek externally derived comfort and control (Conger et al., 2002; Needlman, 2011). When parenting stress compromises parents’ emotion regulation and patience, relational friction may become increasingly unavoidable, with consequent reductions in children’s emotional security (Deater-Deckard and Petrill, 2004). In the present study, children in high-conflict family contexts were observed to be more likely to treat digital media as a form of “psychological refuge” (Hale and Guan, 2015). This pattern suggests that children’s PMU may not necessarily reflect a deliberate preference, but may instead represent a passive, compensatory adaptation to stress exposure within conflictual family environments.

Finally, the most informative finding concerned the serial indirect effect through parental phubbing and parent–child conflict in the association between parenting stress and young children’s PMU. This result underscores the importance of the quality and continuity of family interactions for children’s media use behaviors. Under heightened stress, mobile devices may be relied upon for short-term alleviation of emotional distress (McDaniel and Radesky, 2020). However, when parents are repeatedly absorbed in their phones during parent–child interactions, children’s emotional needs may fail to be responded to in a timely manner, which may elicit perceived neglect or rejection (Zhang et al., 2023). Such affective frustration is likely to provoke negative child responses (e.g., crying, resistance, persistent bids for attention), while parents under stress may lack the patience required for sensitive responding; consequently, interactions may escalate into parent–child conflict (Kildare and Middlemiss, 2017). Collectively, these findings suggest that the association between parenting stress and children’s media use should not be reduced to a narrow issue of emotion management, but should be understood as being deeply embedded in the quality of parent–child interaction processes (Wong et al., 2020). From a family systems perspective, this framework highlights how interactional attunement may shape children’s media dependency, and converging evidence has suggested that harmonious parent–child relationships may buffer stress and reduce children’s reliance on media (Domoff et al., 2019).

### 5.2 Practical implications

This study holds important practical implications, particularly for assisting families in managing media use and improving parent–child relationships. First, the critical role of parenting stress in children’s PMU should not be overlooked. The findings highlight that high levels of stress serve as a common root cause of both negative coping strategies (such as parental phubbing) and increased family conflict. Parents should learn to regulate their emotional stress and avoid adopting negative parenting behaviors driven by excessive pressure. To achieve this, parenting responsibilities should be reasonably shared among family members to reduce the burden on a single parent. Support from relatives, friends, or broader social networks should also be sought to alleviate daily and parenting-related stress. Additionally, parents may consider regular psychological counseling to enhance emotional regulation skills. Such efforts not only reduce stress-induced emotional fluctuations but also strengthen parents’ patience and emotional stability in the parenting process. Emotional regulation training enables parents to better manage their feelings and decreases the tendency to use media as a means of avoiding parent–child interactions (Crnic et al., 2005; McMahon and Meins, 2012).

Second, parents should enhance their media literacy and learn effective strategies for managing media use within the family, thereby preventing digital devices from becoming distractions in parent–child interactions. Specifically, parents can establish “no-phone” periods during key family moments—such as mealtimes, playtime, and before bedtime—to foster high-quality interaction. These periods should be completely free of phone or electronic device use, allowing parents to focus on direct communication and shared activities such as storytelling, art creation, or parent–child games. Research indicates that parents who reduce phone use improve the quality of parent–child relationships and serve as healthy role models for media use, thereby reducing children’s dependence on digital media (Radesky et al., 2015; Domoff et al., 2018). Moreover, parents can collaboratively establish family media use rules with their children, ensuring consistent adherence while making adjustments when necessary to maintain a supportive environment for healthy development.

Finally, parents should focus on improving communication within the family, particularly by establishing positive and effective parent–child communication mechanisms. By practicing active listening and nonviolent communication skills, parents can better understand their children’s emotional needs and avoid negative responses such as scolding or neglect under stressful circumstances. Positive communication reduces parent–child conflict, enhances children’s emotional security, and fosters relational harmony. In addition, parents should work with their children to establish healthy media use guidelines and encourage constructive emotion regulation strategies. For example, parents can guide children to manage emotions through exercise, art, or social engagement, thereby decreasing reliance on media as a coping tool for emotional deprivation. Through these practices, parents can effectively reduce media dependency arising from parent–child conflict or emotional neglect (Gottman et al., 2015; Domoff et al., 2020).

### 5.3 Limitations and future research

Several limitations of the present study point to avenues for future research. First, despite the temporal separation of study variables via a multi-wave design, the absence of baseline controls for the outcome variable precludes strict causal inferences. Future investigations should utilize full longitudinal designs with cross-lagged panel analysis or experimental manipulations to rigorously validate the proposed causal pathways. Second, all data were collected via parental self-reports, which may introduce social desirability bias. Given the sensitive nature of behaviors such as parental phubbing and parent–child conflict, parents might inadvertently underreport the frequency or severity of these interactions. Future studies would benefit from incorporating multi-informant approaches (e.g., spousal evaluations or teacher reports) and objective screen-time tracking applications to enhance measurement validity. Third, the reliance on a sample from families with children attending urban kindergartens in Guangdong Province, China, may limit the generalizability of the findings. Subsequent studies are encouraged to test the model’s stability in broader cultural and regional contexts. Fourth, regarding variable scope, potential omitted variable bias cannot be ruled out. Unmeasured factors, such as parental emotional regulation strategies and the quality of parent–child attachment, may also play important roles in shaping children’s media use. Future research should include more diverse psychological and relational variables to provide a more comprehensive understanding of these mechanisms. Finally, given the focus on traditional smart devices, the impact of rapidly evolving digital tools remains unaddressed. Future research should examine the effects of emerging technologies, such as AI-based interactive devices and virtual parenting assistants, on parent–child interactions and children’s psychological development.

## 6 Conclusion

This study was conducted within the framework of family systems theory and the family stress model to examine the chained mediation through which parenting stress influences young children’s PMU via family interaction mechanisms. It was demonstrated that parenting stress directly predicts children’s PMU. More importantly, a cascade effect was identified through a deteriorating family interaction pathway. Elevated parenting stress was found to significantly increase parental phubbing. This interactional disruption was subsequently shown to exacerbate parent–child conflict, which ultimately led to increased levels of PMU in young children. The primary contribution of this research lies in revealing how parenting stress, as a form of individual psychological distress, is transmitted through the serial pathway of “parental phubbing” (a media-specific behavior) and “parent–child conflict” (a form of family dysfunction), ultimately spilling over and manifesting as externalizing behavioral problems in young children.

## Data Availability

The original contributions presented in the study are included in the article/supplementary material, further inquiries can be directed to the corresponding author.
